# Hypoxia drives glucose transporter 3 expression through hypoxia-inducible transcription factor (HIF)–mediated induction of the long noncoding RNA NICI

**DOI:** 10.1074/jbc.RA119.009827

**Published:** 2019-11-05

**Authors:** Victoria Lauer, Steffen Grampp, James Platt, Veronique Lafleur, Olivia Lombardi, Hani Choudhry, Franziska Kranz, Arndt Hartmann, Bernd Wullich, Atsushi Yamamoto, Mathew L. Coleman, Peter J. Ratcliffe, David R. Mole, Johannes Schödel

**Affiliations:** ‡ Department of Nephrology and Hypertension, Universitätsklinikum Erlangen and Friedrich-Alexander-Universität Erlangen-Nürnberg, Ulmenweg 18, 91054 Erlangen, Germany; § NDM Research Building, University of Oxford, Old Road Campus, Headington, Oxford OX3 7FZ, United Kingdom; ¶ Department of Biochemistry, Faculty of Science, Center of Innovation in Personalized Medicine, King Fahd Center for Medical Research, King Abdulaziz University, Jeddah, Saudi Arabia; ‖ Department of Computer Science 9, Friedrich-Alexander-Universität Erlangen-Nürnberg, Cauerstraße 11, 91058 Erlangen, Germany; ** Institute of Pathology, Universitätsklinikum Erlangen and Friedrich-Alexander-Universität Erlangen-Nürnberg, Krankenhausstraße 8–10, 91054 Erlangen, Germany; ‡‡ Department of Urology and Pediatric Urology, Universitätsklinikum Erlangen and Friedrich-Alexander-Universität Erlangen-Nürnberg, Krankenhausstraße 12, 91054 Erlangen, Germany; §§ Institute of Cancer and Genomic Sciences, University of Birmingham, Edgbaston, Birmingham B15 2TT, United Kingdom

**Keywords:** hypoxia, hypoxia-inducible factor (HIF), glucose transport, long noncoding RNA (long ncRNA, lncRNA), ChIP-sequencing (ChIP-Seq), GLUT3, ChIP

## Abstract

Hypoxia-inducible transcription factors (HIFs) directly dictate the expression of multiple RNA species including novel and as yet uncharacterized long noncoding transcripts with unknown function. We used pan-genomic HIF-binding and transcriptomic data to identify a novel long noncoding RNA Noncoding Intergenic Co-Induced transcript (NICI) on chromosome 12p13.31 which is regulated by hypoxia via HIF-1 promoter-binding in multiple cell types. CRISPR/Cas9-mediated deletion of the hypoxia-response element revealed co-regulation of NICI and the neighboring protein-coding gene, solute carrier family 2 member 3 (*SLC2A3*) which encodes the high-affinity glucose transporter 3 (GLUT3). Knockdown or knockout of NICI attenuated hypoxic induction of SLC2A3, indicating a direct regulatory role of NICI in SLC2A3 expression, which was further evidenced by CRISPR/Cas9-VPR–mediated activation of NICI expression. We also demonstrate that regulation of *SLC2A3* is mediated through transcriptional activation rather than posttranscriptional mechanisms because knockout of NICI leads to reduced recruitment of RNA polymerase 2 to the *SLC2A3* promoter. Consistent with this we observe NICI-dependent regulation of glucose consumption and cell proliferation. Furthermore, NICI expression is regulated by the von Hippel–Lindau (VHL) tumor suppressor and is highly expressed in clear cell renal cell carcinoma (ccRCC), where SLC2A3 expression is associated with patient prognosis, implying an important role for the HIF/NICI/SLC2A3 axis in this malignancy.

## Introduction

Hypoxia is a critical feature of many physiological and pathophysiological conditions (1). Hypoxia-inducible transcription factors (HIFs)^2^ are a major component of the transcriptional response to oxygen deprivation (2). Upon hypoxic exposure HIF-α subunits (HIF-1α and HIF-2α) are stabilized in the cell, following which they dimerize with HIF-1β and translocate into the nucleus to stimulate transcription of genes relevant for multiple regulatory pathways involved in cellular and organismal adaption to the hypoxic insult (*e.g.* metabolism, cell cycle, red blood cell production, and angiogenesis) (3, 4). The HIF pathway can be activated in all tissues and is currently exploited pharmacologically in patients with chronic kidney disease to increase erythropoietin production (5). Beside their physiological functions, HIFs are also important modulators of several human diseases and associated pathological processes, including tumorigenesis (6). The direct relevance of HIFs for cancer progression has been most clearly demonstrated in clear cell renal cell carcinoma (ccRCC), which in most cases is caused by loss of the von Hippel-Lindau (VHL) tumor suppressor (7–10). VHL-dependent ubiquitination is necessary for proteasomal degradation of the HIF-α subunits in normoxic conditions. Therefore, dysfunctional VHL leads to stabilization of HIFs irrespective of oxygen availability, thereby contributing to the development and progression of renal cancer (11).

HIFs can activate the expression of a multitude of metabolic enzymes and transporters to optimize energy production in hypoxia (1, 12). Together, this HIF-mediated transcriptional reprogramming of metabolism supports a shift toward anaerobic energy production. For example, increased glycolysis during hypoxia is supported by HIF-mediated induction of glucose transporters, including solute carrier family 2 member 1 (*SLC2A1*, coding for glucose transporter 1 (GLUT1)) and *SLC2A3* (coding for GLUT3) (13, 14). Although the overall increase in expression of *SLC2A1* and *SLC2A3* by hypoxia and HIF is well-documented, detailed mechanisms of transcriptional regulation of these transporter genes by HIF are less well-defined (13–17).

The repertoire of protein-coding genes activated by HIF has been studied extensively by transcriptome analyses in a variety of cellular settings and, beside a small number of ubiquitous HIF targets (including *SLC2A3*), displays marked cell type specificity (15). Recent work suggests that HIFs also dictate the expression of several classes of nonprotein coding genes, such as micro-RNA and long noncoding RNAs (18). For example, the micro-RNA mir210 has been identified as a direct target of HIF, and its expression is associated with poor or favorable prognosis, respectively, depending on the type of cancer examined (19, 20). The long noncoding RNAs NEAT1 and MALAT1 are also subject to hypoxic regulation and have important roles in tumor biology (21). These findings are in line with recent pan-genomic analyses of transcriptional dysregulation in a variety of diseases which have highlighted the importance of long noncoding transcripts in disease progression (22). However, the potential breadth of long noncoding RNA regulation in hypoxia, and the role of such targets in the hypoxic response, is not yet fully understood.

In recent work, we characterized global HIF-DNA interactions and transcriptional changes mediated via hypoxia in MCF-7 breast cancer cells (4, 18). We observed transcriptional activation of all classes of RNAs by HIF and identified multiple novel and previously nonannotated RNAs with low protein-coding potential. These RNAs were barely detectable in cells exposed to ambient atmospheric oxygen conditions but were highly up-regulated in hypoxia through HIF. Here we investigate the function and regulation of a novel hypoxia-specific long noncoding RNA (NICI) that is directly targeted by HIF. Importantly, we demonstrate that NICI is co-regulated with a neighboring gene, *SLC2A3*. Our analysis revealed a complex and noncanonical regulation of *SLC2A3* expression which is critically dependent on the presence of NICI.

## Results

### HIF controls expression of a promoter-associated long noncoding RNA on chromosome 12p13.31

To gain insights into the regulation of novel transcripts, we intersected existing HIF DNA-binding data in MCF-7 breast cancer cells (400 HIF-1 and 425 HIF-2 binding sites) with 37 loci expressing novel RNAs regulated by hypoxia (4, 18). We showed earlier that all of these transcripts have low protein coding potential (18). Most of the RNAs (*n* = 35) were induced by hypoxia and were at the limit of detection under normoxic conditions. Of the 37 regions expressing novel nonannotated RNAs, 10 were adjacent to HIF-binding events (HIF-1 and HIF-2) within ±10 kb of the putative transcriptional start site (Table S1). These results suggest that HIF binding directly activates transcription of a substantial part of the novel, hypoxia-regulated, nonannotated transcripts in MCF-7 cells.

**Figure 1.**
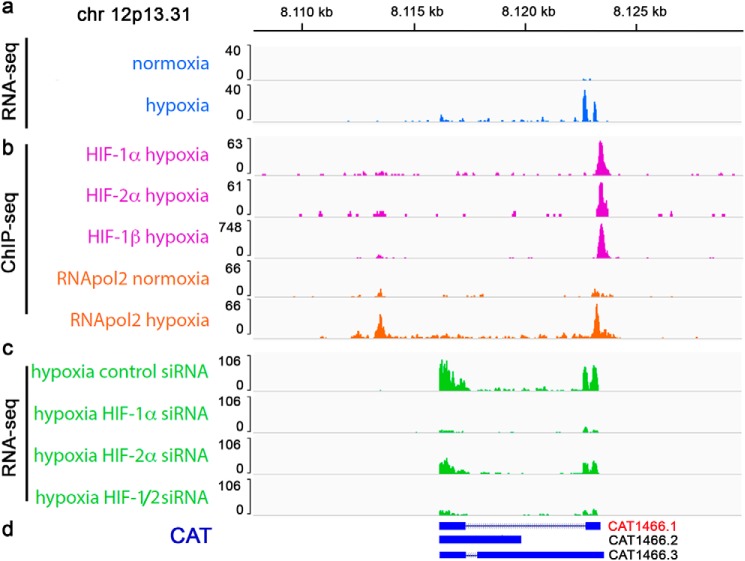
**HIF-1 induces expression of a long noncoding RNA on chr 12p13.31.** *a* and *b*, RNA-Seq tracks (*a*) and ChIP-Seq tracks (*b*) indicate induction of a transcript, HIF binding (HIF-1α, HIF-2α, and HIF-1β) and increased RNA-polymerase 2 activity (RNApol2) in hypoxia at an intergenic site on chr 12p13.31 in MCF-7 breast cancer cells. *c*, RNA-Seq from HIF-siRNA–treated MCF-7 cells cultured in hypoxia reveals a predominant HIF-1 dependence of the long noncoding RNA at this site. *d*, the region overlaps with CAT derived from mitranscriptome.org^3^ (50) from which CAT1466.1 resembles the novel transcript.

One novel transcript with HIF ChIP-Seq signals close to the coding region is located on chromosome 12p13.31 (Fig. 1, *a* and *b*) (18). We used RNApol2 ChIP-Seq in MCF-7 cells cultured under normoxic or hypoxic conditions to assay for transcriptional activity at this locus. The presence of RNApol2 increased in hypoxia, suggesting that elevated levels of the transcript result from *de novo* transcription (Fig. 1*b*). To test for HIF-dependent transcription we interrogated available RNA-Seq data sets from MCF-7 cells cultured in hypoxia with suppressed HIF levels (Fig. 1*c*) (18). Although suppression of HIF-2α had some effect on the hypoxic induction of the transcript, suppression of HIF-1α resulted in a more pronounced reduction of hypoxic expression, indicating that the transcript is predominantly regulated by HIF-1. We also screened existing databases of long noncoding RNAs derived from cancer transcriptomes and discovered that three cancer-associated transcripts (CAT) overlap this region (CAT1466.1, CAT1466.2, and CAT1466.3) (22). From our RNA-Seq data we infer that CAT1466.1 is identical to the transcript which is regulated by HIF (Fig. 1*d*). We further analyzed epigenetic features at the HIF-binding site and detected high levels of the promoter-associated histone mark H3K4me3 and low levels of the enhancer histone mark H3K4me1 in MCF-7 cells (Fig. S1). Moreover, the levels of H3K4me3 and the activity mark H3K27ac increased upon hypoxic exposure indicating that CAT1466.1 is induced from a genuine promoter, rather than expressed as an enhancer-RNA (eRNA) (Fig. S1). This finding is in line with data from genome-wide analyses defining promotor- and enhancer-linked genomic regions which revealed strong evidence for promotor-associated features at this site (23, 24).

### The long noncoding RNA NICI is co-expressed with SLC2A3

**Figure 2.**
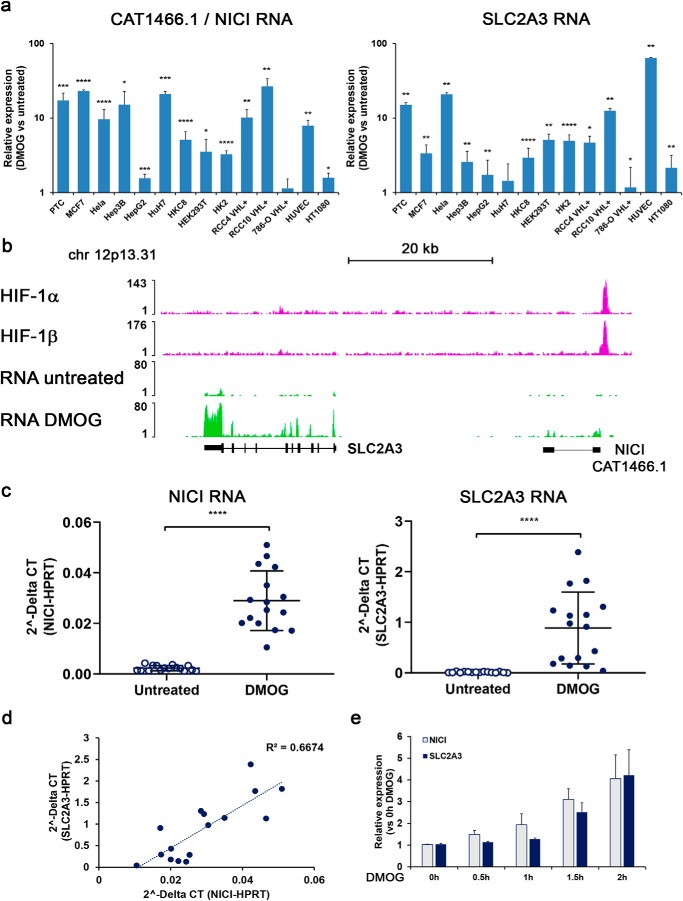
**SLC2A3 and NICI are co-regulated by HIF-1.** *a*, relative NICI and SLC2A3 RNA expression levels (compared with untreated control) in a selection of human cell lines exposed to 1 mm DMOG for 16 h as measured by qPCR (mean ± S.D. from three independent experiments per cell line). Statistical analyses were performed using the one-sample *t* test (*, *p* < 0.05; **, *p* < 0.01; ***, *p* < 0.001; ****, *p* < 0.0001). *b*, ChIP-Seq analysis in PTCs confirms the single HIF-binding site at the CAT1466.1/NICI locus ∼35 kb upstream of *SLC2A3*. Expression of NICI and SLC2A3 is induced upon HIF-stabilization with 1 mm DMOG in RNA-Seq experiments. *c*, mRNA expression of NICI and SLC2A3 in a collection of 16 PTC cultures left untreated or treated with 1 mm DMOG for 16 h. *d*, expression levels of NICI correlate well with those of SLC2A3 in DMOG-treated PTC. *e*, time course of relative expression levels of NICI and SLC2A3 RNA in PTC exposed to 1 mm DMOG for the indicated time. Data are normalized to time point 0 h and *bars* represent mean ± S.D. measured in cells from *n* = 3 individuals.

To date the function of long noncoding RNAs remains poorly understood. However, a number of long noncoding RNAs have a role in the cis-regulation of neighboring coding genes (25). We therefore determined the extent of regulation of CAT1466.1 compared with *SLC2A3*, which is the closest annotated gene ∼35 kb downstream of CAT1466.1. We exposed a broad selection of different human cell lines to the HIF stabilizer dimethyloxalylglycine (DMOG) or 1% hypoxia for 16 h and measured expression of SLC2A3 and CAT1466.1 by quantitative PCR (qPCR) (Fig. 2*a* and Fig. S2). We detected significant and in general comparable levels of induction of SLC2A3 and CAT1466.1 by DMOG or hypoxia, respectively. Increased expression for both genes was observed in most cells examined except for 786-O VHL re-expressing cells, which lack functional HIF-1α (9, 26). Given the striking co-regulation, we suggested the name Noncoding Intergenic Co-Induced transcript (NICI) for the long noncoding RNA CAT1466.1. Consistent with an important role of HIF-1α, HIF knockdown experiments confirmed HIF-1α as the main inducer of both NICI and SLC2A3 in a subset of tested cell lines (Fig. S3, *a–c*). To verify the association of NICI and SLC2A3 expression with HIF binding across cell types, we inspected available HIF ChIP-Seq and RNA-Seq data from additional cell lines (primary renal tubular cells (PTC), RCC4, HKC-8, T47D) for signals in this region. These analyses confirmed a single HIF-binding site in the broader genomic region of *SLC2A3* which corresponded to the *NICI* promoter and regulation of both genes by hypoxia (Fig. 2*b* and Fig. S4). We also examined for regulation of additional neighboring mRNA transcripts (within 1Mb of the novel locus) by hypoxia in available RNA-Seq data sets, but did not detect hypoxic regulation of any other gene in this region (data not shown). We proceeded to analyze this co-regulation in more detail: Expression levels of NICI and SLC2A3 were highly induced in primary renal tubular cell cultures (*n* = 16) treated with DMOG (Fig. 2*c*). Furthermore, NICI RNA levels correlated well with those of SLC2A3, consistent with a shared mode of transcriptional activation (Fig. 2*d*). In this respect we considered the existence of a common RNA from which both transcripts arise, *e.g.* through alternative promoter usage. However, we could not detect the presence of RNApol2 or any transcript in the intergenic region between SLC2A3 and NICI (Fig. 2*b* and Fig. S4) (data not shown). We were also unable to detect spliced RNA products which cover both transcripts by PCR (data not shown). In addition, in time-course experiments we measured a delayed hypoxic induction of SLC2A3 mRNA compared with NICI RNA (Fig. 2*e*). This indicates that NICI and SLC2A3 expression is regulated from autonomous promoters. Our data suggest that hypoxic regulation of *NICI* and *SLC2A3* is mediated via HIF binding to a single regulatory element which is conserved across the cell lines tested.

### Regulation of NICI in renal cancer

**Figure 3.**
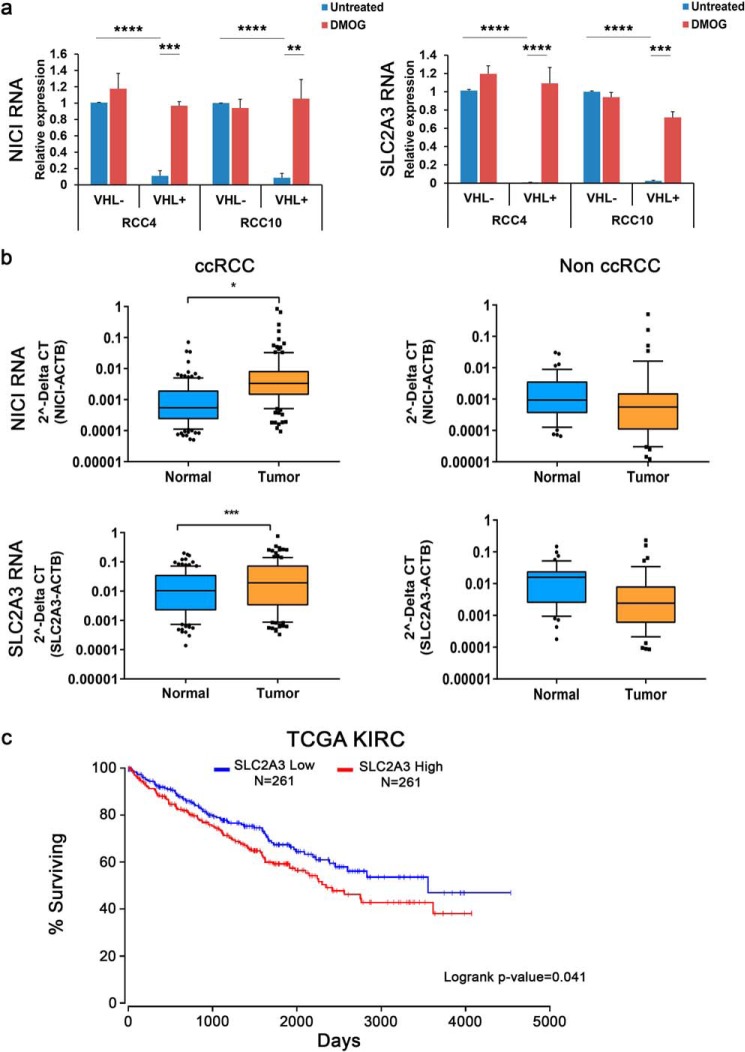
**Expression of NICI and SLC2A3 in renal cancer.** *a*, NICI and SLC2A3 are regulated via VHL and HIF. Expression qPCR analysis of renal cancer cell lines RCC4 VHL ± and RCC10 VHL ± were treated with 1 mm DMOG for 16 h or left untreated. Values are mean ± S.D. from three independent experiments. *b*, box-and-whisker plot of RNA expression levels of NICI and SLC2A3 in samples from ccRCC (*n* = 126) and non-ccRCC (*n* = 35) from the Erlangen RCC cohort compared with corresponding normal renal tissue. Values were measured by qPCR and normalized to the expression levels of actin B (*ACTB*). The *horizontal lines* represent the average value, and the *whiskers* extent to the 10th and 90th percentile, respectively. Statistical analyses were performed using the two-sample *t* test (*, *p* < 0.05; **, *p* < 0.01; ***, *p* < 0.001; ****, *p* < 0.0001). *c*, Kaplan-Meier survival curve of renal cancer patients from the TCGA kidney renal clear cell carcinoma cohort stratified for high (*red*) or low (*blue*) SLC2A3 expression. Data were derived from oncolnc.org^3^ (49).

Following up on the evidence for co-regulation of *NICI* and *SLC2A3* by HIF, we also examined regulation of these genes in ccRCC, in which HIF target genes are commonly up-regulated. We measured expression levels of both genes in VHL-deficient (high levels of HIF) and VHL–re-expressing (low levels of HIF) renal cancer cell lines (RCC4 and RCC10) exposed to control or DMOG conditions (Fig. 3*a*). Here, we determined a significant reduction of both SLC2A3 and NICI RNA levels in cells re-expressing VHL compared with VHL-deficient cells. The expression of both genes was restored in VHL re-expressing cells following HIF stabilization by DMOG, thus confirming the regulatory role of the VHL/HIF-axis in NICI and SLC2A3 expression.

Next, we proceeded to determine the RNA levels of NICI and SLC2A3 in 161 renal tumor and corresponding normal kidney samples collected from RCC patients from the Erlangen RCC cohort (126 ccRCC and 35 non-ccRCC patients including papillary and chromophobe RCCs) (28). NICI and SLC2A3 RNA were significantly up-regulated in tumor samples from ccRCC patients, but not in nonclear cell tumors, supporting the strong association with the HIF pathway (Fig. 3*b*). These results were also observed in data from the TCGA database: Expression levels of SLC2A3 and NICI (CAT1466.1) were highest in ccRCC samples when compared with levels from other tumor entities available in the database (Fig. S5). Interestingly, in the TCGA kidney renal clear cell carcinoma cohort high SLC2A3 mRNA expression was associated with an adverse prognosis (Fig. 3*c*). Taken together, our data from renal tumor cells and patients as well as data from the TCGA database underline the link between NICI/SLC2A3 regulation and the VHL/HIF pathway. The results also point to a relevant role of this regulation for the outcome of renal cancer patients.

### A single HIF-binding site regulates NICI and SLC2A3 expression

**Figure 4.**
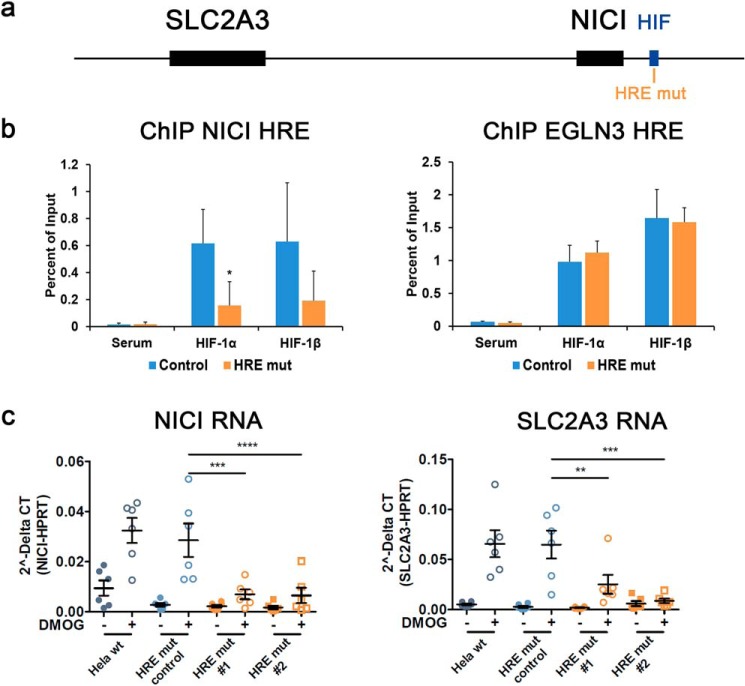
**HIF-binding to the NICI locus regulates SLC2A3 expression.** *a*, the HRE (*HRE mut*) targeted by a specific guide RNA and CRISPR/Cas9-mediated genome editing is highlighted in *orange* in the *NICI* promoter. *b*, HIF-binding to the *NICI* HRE and the control locus *EGLN3* in *NICI* HRE-mutated clones (*n* = 2) and a control clone of cells is shown by ChIP-qPCR (values are from three independent experiments). *c*, relative RNA expression levels of untreated or DMOG (1 mm for 16 h) stimulated HeLa WT cells (*wt*) or clones with (*HRE mut#1* and *HRE mut#2*) or without (*HRE mut control*) mutations in the HRE (*n* = 6 independent experiments). Data represent mean ± S.E. Statistical analyses were performed using the Student's *t* test (*, *p* < 0.05; **, *p* < 0.01; ***, *p* < 0.001; ****, *p* < 0.0001).

To gain more detailed insights into how binding of HIF to a single site on chromosome 12 activates the transcription of two genes we performed CRISPR/Cas9-mediated manipulation of the HIF-binding sequence. We conducted this in HeLa cells which showed consistent induction of both genes by HIF-1 (compare Fig. 2*a* and Fig. S3*c*). The core sequence of the HIF-binding site in the NICI promoter (hg19 chr12:8,123,206–8,123,563 as defined by HIF-ChIP-Seq in MCF-7) contains three hypoxia response elements (HRE) (Fig. S6*a*) (4). We targeted the two HREs in the center of this region in CRISPR/Cas9 experiments (Fig. 4*a* and Fig. S6, *a* and *b*). In HIF-ChIP assays in HeLa cells, mutation of these core HREs significantly reduced HIF binding compared with control clones of cells or WT HeLa cells (Fig. 4*b*) (data not shown). Importantly, these effects were specific because HIF-binding to the *EGLN3* intronic enhancer at chromosome 14 was not affected by mutations at the *NICI* HRE indicating a preserved HIF response at another locus in the mutated cells (Fig. 4*b*). To examine transcriptional consequences of reduced HIF binding to the *NICI* HRE, we determined expression levels of NICI and SLC2A3 in HRE-defective and -nondefective cells upon DMOG exposure. Consistent with their co-regulation, we observed a reduced induction of both transcripts in HRE-mutated cells (Fig. 4*c*). Thus, we have identified a specific HIF-binding DNA element that is capable of regulating expression of two genes, the more proximal long noncoding RNA *NICI* and the more distal glucose transporter *SLC2A3*.

### HIF regulates SLC2A3 expression via induction of NICI

**Figure 5.**
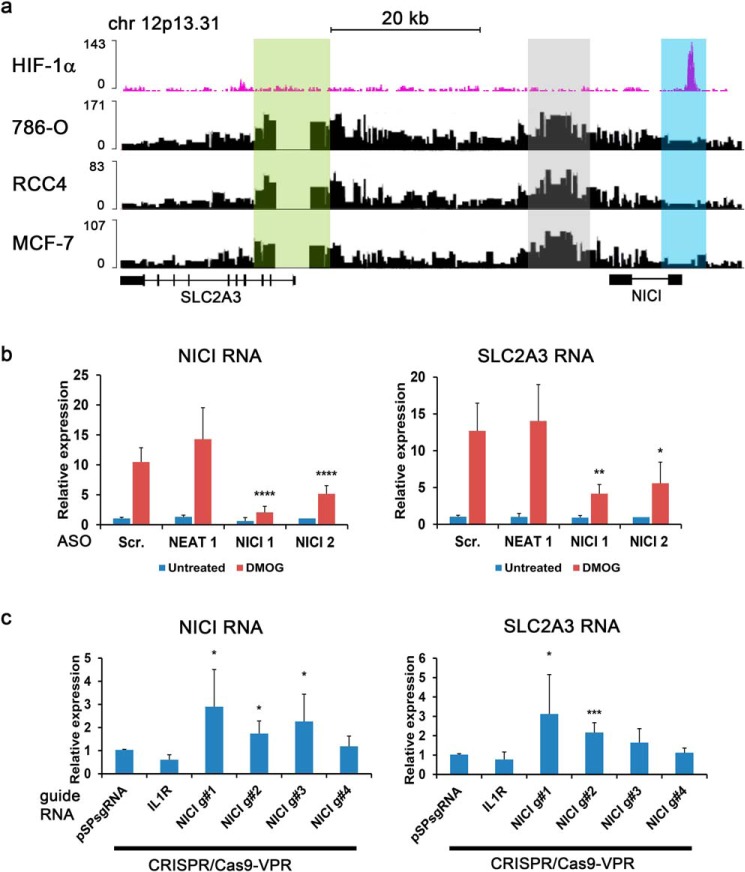
**Activation of NICI expression is necessary for SLC2A3 regulation.** *a*, Capture-C assay reveals chromatin interaction between the *SLC2A3* promoter (anchor site, highlighted in *light green*) and an intergenic site ∼24 kb upstream of the promoter (highlighted in *gray*) in 786-O, RCC4, and MCF-7 cells. No interactions were detected with the *NICI* promoter (highlighted in *light blue*). *b*, knockdown of NICI in HeLa cells using two different ASO targeting NICI (NICI 1 and NICI 2), a nontargeting scrambled control (*Scr*.) and an ASO targeting the long noncoding RNA NEAT1 (*n* = 6 independent experiments). Expression qPCR from DMOG-treated (1 mm for 16 h) and untreated cells was performed for NICI and SLC2A3 RNA. Values were normalized to Hypoxanthine-guanine phosphoribosyltransferase (HPRT) and untreated scrambled control samples. *c*, epigenetic modulation of the *NICI* promoter by the activator CRISPR/Cas9-VPR leads to increased NICI and SLC2A3 expression (*n* = 6 independent experiments). Four different guide RNAs against the *NICI* promoter, pSPsgRNA empty vector or an independent guide RNA targeting the IL1R promoter were used. Values were normalized to results from pSPsgRNA control samples. Statistical analyses were performed using the one-sample *t* test (*, *p* < 0.05; **, *p* < 0.01; ***, *p* < 0.001; ****, *p* < 0.0001).

HIF is able to regulate genes with transcriptional start sites that lie at distances varying from a few base pairs to several hundred kilobases away from the binding site and that are brought together through chromatin looping (4, 29). Therefore, the single HIF-binding site on chr12p.13.31 could transactivate both *NICI* and *SLC2A3* independently. Alternatively, the regulation of either *NICI* or *SLC2A3* could be dependent upon regulation of the other. To test this, we first looked for physical interaction of the *SLC2A3* promoter with the HIF-binding site at the putative *NICI* promoter. We performed chromatin capture assays in three different cell lines (786-O, RCC4, and MCF-7). We used sequences in the *SLC2A3* promoter as anchor sites and examined possible interactions with any distant chromatin region using the Capture-C method (Fig. 5*a*) (29, 30). Although we determined interactions with a putative enhancer site ∼24 kb upstream of the *SLC2A3* promoter, no interaction was observed with the HIF-binding site, which regulates *NICI* and *SLC2A3* expression. This finding suggests that induction of SLC2A3 expression by HIF is not initiated through direct interaction between the *SLC2A3* promoter and the HIF-binding site at the *NICI* promoter (*i.e.* acting as a distant enhancer of *SLC2A3*) but may instead be mediated via the induction of NICI and subsequent regulation of *SLC2A3* transcription.

Long noncoding RNAs can be involved in transcriptional regulation of neighboring genes by various mechanisms, including recruitment of transcriptional modulators (*e.g.* activators or repressors) or direct interaction with the target transcript (31). Despite the lack of direct interaction between the HIF-binding site and the *SLC2A3* promoter the strong association between HIF-binding and expression of both genes as well as the delayed induction of SLC2A3 compared with NICI (Fig. 2*e*) suggests a functional role for NICI in regulating SLC2A3 expression. To validate this hypothesis, we first suppressed the expression of NICI using two different antisense oligonucleotides (ASOs) in HeLa cells. We achieved 80 and 50% knockdown efficiency of NICI in hypoxia with NICI ASO1 and ASO2, respectively (Fig. 5*b*). In line with our hypothesis, depletion of NICI resulted in a comparable reduction of SLC2A3 mRNA expression. Similar results for the effect of NICI knockdown on SLC2A3 mRNA and protein levels were obtained in PTC (Fig. S7). This suggests that NICI expression is necessary for hypoxic induction of SLC2A3. To show that NICI expression is sufficient to induce SLC2A3, we used a CRISPR/Cas9 approach to induce transcription of NICI in a HIF-independent manner. We co-transfected the CRISPR/Cas9-VPR transcriptional activator with four different guide RNAs (gRNAs) targeting the core HIF-binding sequence including the sequence spanning the HRE within the *NICI* promoter into HeLa cells. Increased expression of NICI was observed with three of the four guide RNAs when compared with control guides (Fig. 5*c*). Importantly, these three gRNAs also increased levels of SLC2A3 mRNA, although only two reached statistical significance. Because these experiments were conducted under normoxia, we conclude that activation of NICI transcription is sufficient to induce SLC2A3 expression.

### Knockout of NICI affects RNApol2 recruitment to the SLC2A3 promoter and cell proliferation

**Figure 6.**
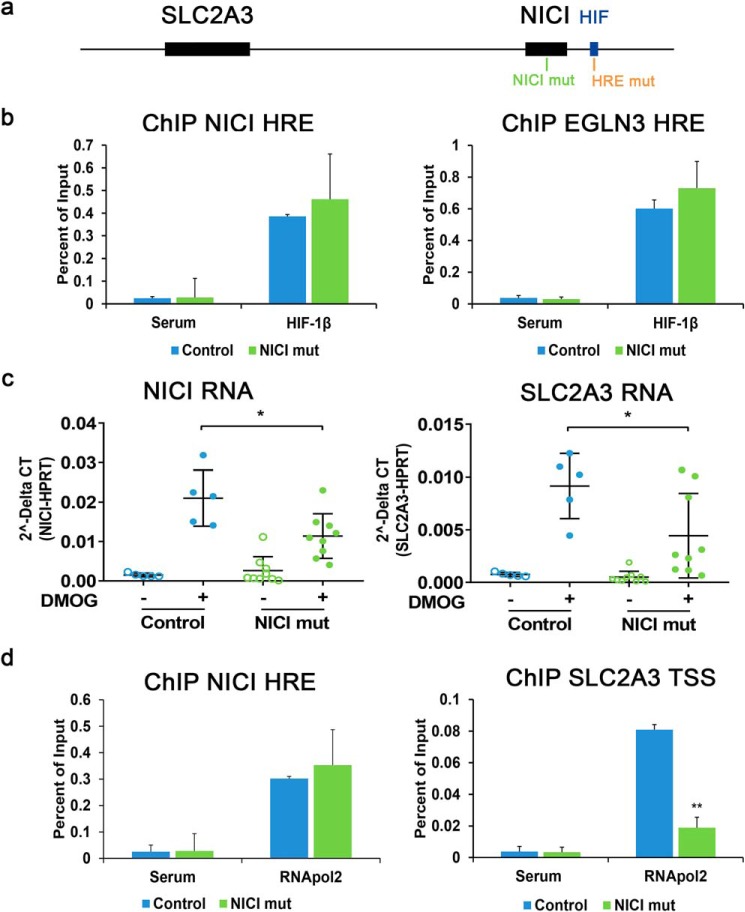
**NICI mediates hypoxic SLC2A3 induction.** *a*, the site within the NICI transcript targeted by the guide RNA for CRISPR/Cas9-mediated mutation is highlighted in *green* (*NICI mut*). *b*, HIF ChIP-qPCR for the *NICI* HRE and the control HRE in the *EGLN3* intronic region. DNA fragments were captured by ChIP using HIF-1β antibodies or serum control in clones with mutations in the NICI transcript (NICI mut) *n* = 4 independent clones) or control clones (control; *n* = 4 independent clones). Cells were exposed to 1 mm DMOG for 16 h. *c*, expression qPCR for NICI and SLC2A3 in untreated or DMOG-treated clones with (NICI mut; *n* = 9 independent clones) or without mutations in the NICI transcript (control; *n* = 5 independent clones). Results are from three independent experiments for each clone of cells. *d*, RNA polymerase 2 (RNApol2) DNA interactions were determined by ChIP qPCR at the *NICI* HRE and the *SLC2A3* transcriptional start site (*TSS*) in NICI transcript-mutated clones (*n* = 4) compared with control clones (*n* = 4). Cells were exposed to 1 mm DMOG for 16 h.

**Figure 7.**
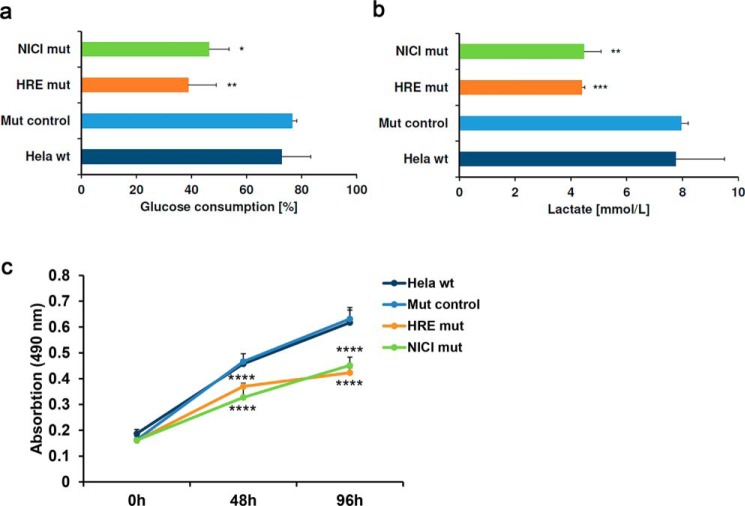
**NICI regulates glucose consumption and proliferation.** *a* and *b*, glucose consumption (*a*) and lactate (*b*) in culture media from hypoxic cells. Cells (*n* = 2 for *HRE mut*, *n* = 3 for *NICI mut*, *n* = 4 for *mut control*) were cultured in 1 mm DMOG for 24 h before glucose consumption and lactate were measured in the medium. *c*, MTS proliferation assay with cells from *a* performed in triplicates per time point under hypoxic conditions (1% O_2_). Statistical analyses were performed using the one- or two-sample *t* test (*, *p* < 0.05; **, *p* < 0.01; ***, *p* < 0.001; ****, *p* < 0.0001) comparing values from NICI/HRE mutated cells to the respective values from control clones (mut control).

To further dissect the mechanism of hypoxic SLC2A3 regulation and the role of NICI, we performed CRISPR/Cas9-mediated deletion of the NICI transcript in HeLa cells (Fig. 6*a*). To minimize effects on transcription factor binding at the *NICI* promoter, we targeted a region ∼330 bp downstream of the functional HRE and outside any potential transcription factor–binding sites as detected in Encyclopedia of DNA Elements analyses (Fig. S6*c*) (data not shown). Nine clones harboring mutations in the *NICI* coding region that did not affect the core HRE or other putative regulatory regions were generated (Fig. S6*c*). Importantly, mutations in the gene body of *NICI* did not alter HIF binding to the *NICI* HRE or the *EGLN3* HRE (Fig. 6*b*). However, mutation of *NICI* led to significantly reduced induction of both NICI and SLC2A3 RNA by DMOG when compared with control clones (Fig. 6*c*). To examine whether the regulation of *SLC2A3* by NICI is mediated directly through transcriptional activation, we performed RNApol2 ChIP under DMOG treatment (Fig. 6*d*). As expected from the HIF-ChIP experiments (Fig. 6*b*), RNApol2 was recruited to the HIF-binding site in the *NICI* promoter following DMOG treatment, which was unaffected by mutations in the *NICI* gene. Importantly however, recruitment of RNApol2 to the *SLC2A3* promoter by DMOG was significantly reduced in *NICI*-mutated clones, indicating a direct role of NICI in transcriptional activation of SLC2A3 expression (Fig. 6*d*). Finally, to test for functional consequences of HRE and *NICI* mutations in metabolic and growth responses we measured glucose consumption, lactate production, and proliferation in hypoxia (Fig. 7, *a–c*). Interestingly, mutations in either the *NICI* HRE or the *NICI* gene body caused a remarkable reduction in glucose consumption, lactate production, and proliferation in HeLa cells under hypoxic conditions. Importantly, hypoxic induction of SLC2A1 (GLUT1), the other glucose transporter targeted by HIF, was not compromised by mutations in the *NICI* gene (Fig. S8). This suggests that binding of HIF at the *NICI* locus and activation of NICI is important for cell growth under hypoxia and that this effects is mediated by regulation of the SLC2A3 isoform of glucose transporters.

Taken together, our experiments reveal the presence of a novel transcript we term NICI, which is transcriptionally activated by the HIF-1 transcription factor. An intact NICI transcript is both necessary and sufficient for the hypoxic transcriptional activation of *SLC2A3* by HIF and is involved in regulating glucose consumption and cell proliferation.

## Discussion

HIFs coordinate a complex transcriptional program that drives cell adaption to hypoxia. Regulation of established protein-coding genes has been investigated in detail in single gene analyses or genome-wide approaches. These studies revealed that HIFs mainly act in cis by occupying promoter-proximal or promoter-distal regulatory elements to induce gene expression (3, 32, 33). In this respect, we have previously shown that HIF frequently binds to enhancers and that gene regulation may occur through looping of these distant sites to target promoters (28, 29, 34). Here we describe an alternative mechanism by which HIFs may regulate distant genes in trans through induction of a long noncoding RNA. Our experiments using ChIP- and RNA-Seq, chromatin capture assays, coupled with genome editing and artificial transcriptional activators in a variety of primary and transformed cell models identify the promoter-associated long noncoding RNA NICI and describe its functional role for hypoxic SLC2A3 regulation.

Local control of gene transcription has been described as one of the main functions of long noncoding RNAs (31). Recent reports have highlighted this way of fine-tuning transcriptional activity with important relevance for human diseases. For example, in a recent genome-wide screen testing resistance to BRAF inhibitors in melanoma cells, CRISPR/Cas9-mediated activation of more than 10,000 lncRNAs revealed 16 candidate loci conferring resistance to BRAF inhibition (25). Importantly, analysis of a subset of these lncRNA showed that activation of lncRNAs can lead to differential transcriptional activity of protein-coding genes in genomic vicinity (within 1 Mb) of the respective lncRNA locus. These findings are in accordance with our results obtained in experiments with CRISPR/Cas9-VPR–activated NICI expression, which led to increased SLC2A3 RNA levels.

The HIF-binding site in the *NICI* promoter was identified in an earlier report using HIF ChIP-Seq in human umbilical vein endothelial cells (35). The authors also detected regulation of *SLC2A3* and attributed enhancer-like functions to the HIF-binding site using 3C and reporter assays. Using Capture-C assays, we did not detect chromatin looping of the *SLC2A3* promoter to the HIF-binding site within the *NICI* locus in three different cell lines. The discrepancy between the two studies might be caused by the different methods applied or by the different cell lines used having potentially diverse chromatin configurations in this region. We detected chromatin interactions of the *SLC2A3* promoter with a genomic region ∼24 kb upstream of the *SLC2A3* gene and 10 kb downstream of *NICI*. This site has been described to interact with the *NICI* locus and may therefore mediate the effect of NICI on SLC2A3 (35).

Both HIF-binding and expression of long noncoding RNAs can vary considerably between cell types. Therefore, the conserved regulation of both *SLC2A3* and *NICI* by HIF across many cell types is noteworthy. Indeed, this may have contributed to the detection of the transcript and the regulatory loop linking NICI to SLC2A3 expression in the current work. Of note, and in contrast to SLC2A1 and most glycolytic genes which are regulated by HIF-1 in many species including humans and mice, SLC2A3 is not regulated by loss of VHL in the renal epithelium of mice (Fig. S9). Consistent with this and in line with the poor conservation of the noncoding transcriptome the *NICI* locus is not conserved in mice (data not shown) (36). This suggests either that NICI expression is species specific or that it is expressed from a different locus and/or in a different form in other species. Therefore, NICI may contribute to fine-tuning of hypoxic SLC2A3 expression specifically in humans.

We also considered the possibility of an alternative promoter usage for SLC2A3 expression at this site. However, we could not detect spliced RNA products that cover both the *NICI* coding region and fragments of the SLC2A3 transcript in PCR analyses (data not shown). Furthermore, we could not detect RNApol2 binding or transcripts in the putative intronic region covering the 35 kb from the *NICI* promoter to the *SLC2A3* gene in any of our sequencing experiments. Confirming our observations, recent studies describe this locus as a region resembling features of a promoter-associated long noncoding RNA (23, 24). We conclude that NICI is a genuine long noncoding RNA with low protein coding potential.

Glucose transporters have a remarkable tissue-specific expression pattern (37). For example, SLC2A3/GLUT3 is mainly detectable in the brain and the testis (38). Earlier studies together with our mRNA expression analyses and a meta-analysis of hypoxia-inducible genes suggest that expression of SLC2A3/GLUT3 is induced in tumor cells and under control of many tumor-associated pathways including the HIF-pathway (15, 39). These observations would be in agreement with an important role of this high-affinity glucose transporter in securing energy supply and cell survival in hypoxic areas of certain tumors that have outgrown sufficiently oxygenated regions. Because fast growth is a feature of many aggressive tumors, increased expression of glucose transporters is associated with poor survival in many cancers (39). Therefore, pharmacological targeting of expression or function of glucose transporters is a promising strategy in oncology research (40). A recent study has highlighted this approach in glioblastoma patients in which high GLUT3 expression associates with an unfavorable outcome (41). Targeting cells which were dependent on GLUT3 by interfering with integrin and PAK4-YAP/TAZ signaling reduced GLUT3 expression and tumor cell viability, introducing a novel approach to treat this aggressive tumor type (41).

Further experiments will be necessary to uncover the detailed mode of action of NICI on SLC2A3 expression. The mechanism might involve direct recruitment of members of the transcriptional machinery or other transcription factors to the *SLC2A3* promoter or the enhancer site interacting with the promoter. It is possible that NICI is involved in modulating the epigenetic signature at the *SLC2A3* gene to permit increased transcription. It will also be of interest to examine the function of NICI in diseases associated with hypoxia signaling such as ccRCC. Furthermore, a detailed analysis of the noncoding transcriptome regulated by hypoxia in different cells and settings might reveal more such loci with co-regulation of lncRNAs and protein-coding genes maybe in a more cell type–specific fashion.

## Experimental procedures

### Cell culture

Human PTCs were obtained from the healthy kidney cortical tissue of patients undergoing tumor nephrectomy. Isolation of these cells was approved by the local ethical committee at the University Erlangen-Nürnberg. Investigations were performed in accordance with the principles of the Declaration of Helsinki. HKC-8 cells were from L. Racusen. 786-O pVHL re-expressing cells were a gift of W. G. Kaelin, Jr. RCC4 cells were provided by C. H. Buys. RCC10 cells were from M. Wiesener. HK-2 and HUH-7 were kindly provided by C. Warnecke. Human umbilical vein endothelial cells were a gift from the Department of Molecular Cardiology, University Erlangen-Nürnberg. HeLa, MCF-7, Hep3B, HepG2, HEK293T, T47D, and HT1080 cells were purchased from American Type Culture Collection. PTCs were cultured in DMEM and Ham's F-12 supplemented with 2 mm l-glutamine, 100 units ml^−1^ penicillin, 100 μg ml^−1^ streptomycin, 5g ml^−1^ insulin, 5 μg ml^−1^ transferrin, 5 ng ml^−1^ selenium (Sigma), 10 ng ml^−1^ tri-jodothronin, 1 mg hydrocortisone, and 100 μg ml^−1^ epidermal growth factor (PeproTech) (42). HKC-8 cells were grown as described previously (43). HT1080 cells were cultured in minimal essential medium supplemented with 10% fetal calf serum, 2 mm l-glutamine, 100 units ml^−1^ penicillin and 100 μg ml^−1^ streptomycin. All other cell lines were grown in DMEM supplemented with 10% fetal calf serum, 2 mm l-glutamine, 100 units ml^−1^ penicillin and 100 μg ml^−1^ streptomycin. Subconfluent cell cultures were exposed to 1 mm DMOG (Cayman Chemical Co.) 16 h before harvest.

### RNA isolation and quantification by qPCR

Total RNA from cell culture was isolated using the peqGOLD Total RNA Kit or TriFast Reagent (VWR peqlab) according to manufacturer's instructions. Transcription of RNA to cDNA was performed using the High-Capacity cDNA Reverse Transcription Kit (Thermo Fisher Scientific). qPCRs were performed using Maxima SYBR Green/ROX qPCR Master Mix (Thermo Fisher Scientific) on a StepOnePlus Real-Time PCR cycler (Applied Biosystems). Primers are listed in Table S2.

### siRNA and ASO transfection

siRNA against Drosophila HIF (control siRNA), HIF-1α, and HIF-2α has been described previously (44) (Table S2). siRNAs were transfected using SAINT red reagent (Synvolux) with a final concentration of 40 nm. Scrambled and NEAT1 ASO controls (45) and NICI ASOs (Integrated DNA Technologies) were transfected using Lipofectamine 3000 reagent (Invitrogen) with a final concentration of 100 nm. ASO sequences are given in Table S2.

### ChIP

ChIP assays were performed using the Upstate Protocol (Millipore) with minor modifications as described (46, 47). Cells were crosslinked by adding 1% (w/v) formaldehyde. Crosslinking was quenched by addition of 125 mm glycine. Cells were scraped off and lysed in 1 ml SDS lysis buffer. Genomic DNA in cell lysates was fragmented using a Bioruptor Plus sonicator (Diagenode). For immunoprecipitations, 6 μl of antibody solutions against HIF-1α (rabbit polyclonal, Cayman Chemicals, Cay10006421) and HIF-1β (rabbit polyclonal, Novus Biologicals, NB100-110) or 10 μl of RNApol2 antibody (Santa Cruz Biotechnology, sc-899) were used. Rabbit serum served as negative control. Chromatin-antibody complexes were pulled down by Protein A agarose beads (Millipore). After reversal of crosslinking by heat, DNA was isolated by phenol-chloroform extraction. Samples were analyzed by ChIP-qPCR using Maxima SYBR green/ROX qPCR Master Mix on a StepOnePlus Real-Time cycler (Applied Biosystems). Primers are listed in Table S2.

### Capture-C assay

Experiments were performed as described previously (29, 30). Briefly, 3C libraries were generated from 786-O, RCC4, and MCF-7 cells with DpnII and were sonicated to 200 bp. Indexed libraries were generated with NEBNext reagents (E6000, E7335, New England Biolabs). Capture enrichment was performed with the SeqCap EZ system (06953212001, Roche/Nimblegen) following the manufacturer's instructions. 1–2 μg of indexed library was incubated with 13 pmol of a pool of biotinylated oligos (Integrated DNA Technologies or Sigma). A double capture protocol was followed with 48-h and 24-h hybridizations (30). Capture efficiency was determined with qPCR relative to a standard curve of genomic DNA prior to sequencing.

### Genome editing

For genome editing, the GeneArt CRISPR Nuclease Vector with OFP Reporter Kit (Invitrogen) was used. The gRNA was designed according to algorithms provided by the Zhang lab. Cloning of CRISPR/Cas9 plasmids followed the manufacturer's instructions. A total of 6 × 10^4^ cells were transfected with 3 μg plasmid using Lipofectamine 3000 (Invitrogen). Single-cell clones were generated by dilution. For mutation screens, genomic DNA of each clone was isolated by phenol-chloroform extraction and the CRISPR/Cas9 target region was amplified by PCR. PCR products were resolved in a 15% nondenaturating polyacrylamide gel. Primers are listed in Table S2. Genomic DNA of clones with putative indel mutations was amplified by PCR, cloned into a pGL3-Basic vector (Promega), and analyzed by Sanger sequencing.

### CRISPR/Cas9 mediated gene activation

For CRISPR/Cas9-mediated activation of gene expression we used the SP-dCas9-VPR artificial activator (VP64-p65-Rta; Addgene 63798) in combination with a pSpgRNA plasmid (Addgene 47108) as a carrier for the specific gRNA (48). The gRNAs were designed according to algorithms provided by the Zhang lab. For cloning of the gRNA into the carrier vector we used the restriction enzyme BbsI according to the manufacturer's instructions. A total of 3 × 10^4^ cells were transfected with 375 ng CRISPR/Cas9-VPR in combination with 125 ng pSPgRNA-Nici guide using Lipofectamine 3000 (Invitrogen). Total RNA was isolated as described 48 h after transfection.

### Immunoblotting

Cells were lysed in Urea/SDS or RIPA buffer added with 1× cOmplete Protease inhibitor (Roche) and 5 mm DTT. Proteins were fractionated by size in 10% (w/v) denaturating SDS-polyacrylamide gels and transferred onto polyvinylidene difluoride membranes. For detection, antibodies anti-HIF-1α (1:1000, rabbit polyclonal, Cayman Chemicals, Cay10006421), anti-HIF-2α (1:1000, goat polyclonal, R&D Systems, AF2997), anti-Glut3 (1:400, rabbit polyclonal, Abcam, ab15311), and anti-β-actin-HRP (1:60,000, mouse monoclonal (AC-15), Sigma-Aldrich, A3854) and horseradish peroxidase–conjugated anti-rabbit (1:1000) and anti-goat (1:1000) secondary antibodies (Dako) were used. Protein signal was detected using Pierce ECL Plus Western blotting substrate (Thermo Fisher Scientific). Immunoreactive bands were quantified using the ImageQuant TL 8.1 software (GE Healthcare) and normalized to signals for β-actin. Specificity of the antibodies was validated using siRNA-mediated knockdown of the respective proteins (for HIF-1α, HIF-2α please compare Fig. S3) (SLC2A3 data not shown).

### Healthy kidney and tumor samples

Healthy human kidney cortical tissue and tumor tissue from patients undergoing tumor nephrectomy was kindly provided by the Comprehensive Cancer Centre (CCC) at the Universitätsklinikum Erlangen. The use of this tissue was approved by the local ethical committee at the University of Erlangen-Nürnberg and each patient gave informed consent. Investigations were performed in accordance with the principles of the Declaration of Helsinki. Tumor and normal kidney samples were examined by an expert pathologist. Pairs of normal kidney and tumor tissue from 126 ccRCC and 35 non-ccRCC patients were used. Fresh frozen tissue was used to isolate total RNA using the peqGOLD Total RNA Kit and expression of genes was measured as described above.

### High-throughput sequencing data

High-throughput sequencing experiments have been performed as described previously (4, 26, 27, 29). Data are available at the GEO and EMBL-EBI Array Express data bases with accession codes GSE78113, GSE120887, GSE28352, GSE101064, GSE54172, E-MTAB-1994, and E-MTAB-1995.

### Proliferation assay

Cells were plated in triplicates at 4 × 10^3^ in 96-well tissue culture plates in 100 μl medium. The next day cells were exposed to 1% O_2_ for 48 or 96 h in a hypoxic incubator. An MTS assay was conducted after 0, 48, or 96 h of hypoxic exposure according to the manufacturer's instructions (Promega). Absorbance was measured in a microplate reader and normalized to background values derived from medium alone.

### Glucose consumption and lactate production

Cells were plated at a density of 8 × 10^4^ in a 6-well plate. The next day cells were incubated with 1 mm DMOG in fresh growth media. After 24 h the supernatant was analyzed for glucose and lactate content using a ABL800 FLEX blood gas analyzer (Radiometer). Values from medium alone (from the beginning of the experiment) were used to calculated percentage of glucose content in the samples.

### Statistical analysis

Statistical analyses were performed using a one-sample, a two-sample, or a paired *t* test as applicable (GraphPad Prism version 8.00 and Microsoft Excel 2016).

## Author contributions

V. Lauer, S. G., J. P., V. Lafleur, O. L., H. C., F. K., A. H., and J. S. data curation; V. Lauer, S. G., J. P., V. Lafleur, B. W., A. Y., M. L. C., and J. S. formal analysis; V. Lauer, S. G., J. P., V. Lafleur, O. L., H. C., A. H., B. W., M. L. C., D. R. M., and J. S. investigation; V. Lauer, P. J. R., D. R. M., and J. S. writing-original draft; F. K. software; F. K. visualization; A. Y., P. J. R., and J. S. methodology; P. J. R., D. R. M., and J. S. funding acquisition; J. S. conceptualization; J. S. supervision.

## Supplementary Material

Supporting Information

## Footnotes

3 Please note that the JBC is not responsible for the long-term archiving and maintenance of this site or any other third party hosted site.

## Footnotes

2 The abbreviations used are:

HIF: hypoxia-inducible factor
ASO: antisense oligonucleotide
CAT: cancer associated transcript
ccRCC: clear cell renal cell carcinoma
DMOG: dimethyloxalylglycine
gRNA: guide RNA
HRE: hypoxia response element
lncRNA: long noncoding RNA
NICI: noncoding intergenic co-induced
PTC: primary tubular cell
TCGA: The Cancer Genome Atlas
VHL: von Hippel-Lindau.
