# Supplementary material for: Hypoxia drives glucose transporter 3 expression through hypoxia-inducible transcription factor (HIF)–mediated induction of the long noncoding RNA NICI
Source: J Biol Chem. 2019 Nov 5;295(13):4065–78. doi: 10.1074/jbc.RA119.009827 (PMC7105321; doi:10.1074/jbc.RA119.009827)
Supplement: Supporting Information [file supp_295_13_4065__index.html]

Hypoxia drives glucose transporter 3 expression through HIF-mediated induction of the long non-coding RNA NICI — Long non-coding RNA NICI regulates GLUT3 expression — Hypoxia drives glucose transporter 3 expression through hypoxia-inducible transcription factor (HIF)–mediated induction of the long noncoding RNA NICI — Long noncoding RNA NICI regulates GLUT3 expression — Supporting Information 

# Hypoxia drives glucose transporter 3 expression through hypoxia-inducible transcription factor (HIF)–mediated induction of the long noncoding RNA NICI

## Supporting Information

- Supporting Information (to be published online) - Supplementary tables and figures
