# Supplementary material for: Hypoxia drives glucose transporter 3 expression through hypoxia-inducible transcription factor (HIF)–mediated induction of the long noncoding RNA NICI
Source: J Biol Chem. 2019 Nov 5;295(13):4065–78. doi: 10.1074/jbc.RA119.009827 (PMC7105321; doi:10.1074/jbc.RA119.009827)

Hypoxia drives glucose transporter 3 expression through HIF-mediated induction of the long non-coding RNA NICI

Victoria Lauer, Steffen Grampp, James Platt, Veronique Lafleur, Olivia Lombardi, Hani Choudhry, Franziska Kranz, Arndt Hartmann, Bernd Wullich, Atsushi Yamamoto, Mathew L Coleman, Peter J Ratcliffe, David R Mole, Johannes Schödel

Supporting information

Supporting information

Supplementary Table 1

Overlap of HIF-bindings sites with loci coding for novel non-coding RNAs detected in hypoxia in MCF-7 breast cancer cells.

Supplementary Table 2

Primers and oligonucleotides used in this study.

Supplementary Figure 1

ChIP-seq analyses of epigenetic features at the HIF-binding site and the locus coding for the novel long non-coding RNA in untreated (normoxia) or hypoxic MCF7 breast cancer cells. These data show high levels of the promotor-associated histone mark H3K4me3 at the HIF-binding site.

Supplementary Figure 2

Relative expression levels of CAT1466.1/NICI and SLC2A3 in a selection of cell lines exposed to 1mM DMOG or 1% hypoxia for 16h. Results are mean ±SD from 2 to 5 independent experiments.

Supplementary Figure 3

siRNA experiments using non-targeting (dHIF), HIF-1α or HIF-2α siRNA transfected into RCC4 VHL+ (a), RCC10 VHL+ (b) or Hela cells (c) (n=3 experiments per cell type). Cells were transfected twice (0 h and 24 h) with the respective siRNA and incubated with 1mM DMOG for 16h or left untreated before harvest at 48 h after the last transfection. NICI and SLC2A3 expression were analysed by qPCR. Representative western blot analyses for HIF-1α and HIF-2α in total protein lysates depict protein status of HIF-1α and HIF-2α, respectively, after siRNA mediated knock-down in all three cell lines. Statistical analyses were performed using the one sample t-test (** $p<0.01$).

Supplementary Figure 4

Snapshot of signals from HIF ChIP-seq experiments (blue) in RCC4 (+VHL), HKC-8 and T47D cells exposed to hypoxia. Experiments identify a single HIF-binding site in the region upstream of *SLC2A3* corresponding to the *NICI* promoter. Corresponding RNA-seq experiments (green) reveal hypoxic induction of both genes.

Supplementary Figure 5

Boxplots comparing **a)** CAT1466.1 and **b)** SLC2A3 expression levels in different TCGA cancer data sets reveal high expression of both genes in clear cell renal carcinoma (ccRCC). Data was derived from mitranscriptome.org (CAT1466.1) or downloaded from the TCGA website (SLC2A3).

Supplementary Figure 6

a) Core HIF-binding sequence in the NICI promoter. HREs are highlighted in red. **b)** and **c)** Sanger sequencing of PCR fragments amplified from DNA isolated from **b)** HRE or **c)** NICI mutated clones and cloned into the pGL3 basic vector. GuideRNAs are indicated in **b)** orange or **c)** green. The targeted HREs are indicated in purple.

Supplementary Figure 7

ASO knock down experiment using non-targeting (scrambled, Scr.) or NICI 2 ASO transfected into PTC (n=1). Cells were transfected twice at 0 h and 24 h with the respective ASO and incubated with 1mM DMOG for 24h or left untreated before harvest at 48 h after the last transfection. **a)** NICI and SLC2A3 expression were analysed by qPCR. **b)** Western blot for GLUT3 in total protein lysates depict protein status with slight reduction of GLUT3 after NICI 2 ASO mediated knock-down in PTC. Pos. ctrl.: Positive control from DMOG treated PTC without ASO.

Supplementary Figure 8

Expression levels of SLC2A1 RNA in Hela HRE (orange), NICI mutated clones (green) or controls (blue). Cells were untreated or exposed to DMOG [1mM] for 16h. Bars show mean ±SD.

Supplementary Figure 9

Expression levels of Egln3, Slc2a1, Slc2a3 RNA in mouse kidneys with or without PAX8-Cre mediated deletion of VHL in renal tubular cells. Bars show mean ±SD. Data was retrieved from GEO data set: GSE54172.

Supplementary Table 1

Number	Chromosome	Start	End	Strand	HIF-1	HIF-2
1	chr1	202053848	202082554	+	yes	yes
2	chr1	202053658	202057903	+	yes	yes
3	chr1	202002600	202088108	+	yes	yes
4	chr1	201983168	202077686	+	yes	yes
5	chr1	181069443	181074240	-		
6	chr1	148241355	148241861	+		
7	chr1	107292908	107293420	+		
8	chr1	107073060	107424139	+		
9	chr1	106734807	106876406	+		
10	chr1	106735450	106762265	+		
11	chr1	45196573	45196842	-		
12	chr10	8911651	8932114	+		
13	chr10	8909994	8931788	+		
14	chr10	5524638	5535702	-		
15	chr11	65222490	65244737	+		
16	chr12	25956136	25959121	+		
17	chr12	8116260	8123253	-	yes	yes
18	chr15	51328348	51690847	+		
19	chr16	85587072	85589839	-		
20	chr16	85495495	85603598	+	yes	yes
21	chr16	85479122	85498916	-		
22	chr16	14395080	14494860	+	yes	yes
23	chr16	85496110	85524891	+		
24	chr2	132300855	132350688	+		
25	chr2	75136093	75145898	+		
26	chr2	1571357	1629598	-	yes	yes
27	chr20	292339	305552	-		
28	chr3	64022004	64104438	+	yes	yes
29	chr5	177485281	177505466	-		
30	chr5	172721232	172730376	+		
31	chr5	102088692	102198533	+		
32	chr5	66509857	66520819	+		
33	chr5	66509437	66891986	+		
34	chr7	157283010	157312766	+	yes	yes
35	chrX	95228382	95229237	-		
36	chr11	70995192	70998931	+		
37	chr7	129244613	129251530	-		

Supplementary Table 2

Expression primer

Primer	5'-3' Sequence
HPRT fw	GACCAGTCAACAGGGGACAT
HPRT rev	AACACTTCGTGGGGTCCTTTTC
NDRG1 fw	CTCTGTTCACGTCACGCTGT
NDRG1 rev	AGAGGGGGTTGTAGCAGGTT
NEAT1-1 fw	CAGTTTTCCGAGAACCAAA
NEAT1-1 rev	ATGCTGATCTGCTGCGTATG
NICI 3' fw	CTCCCTTTCTTGCACTCGTC
NICI 3' rev	GGTAAATGCGTCCAAGAGGA
NICI middle fw	CCCTTCGTCTCTGTTTCTCG
NICI middle rev	CTGAGACCCTGCCTCAAAAG
NICI outside rev.	TTCCAGAGAAGACGCCGATT
SLC2A3 (GLUT3) fw	TCACAGTTGCTACAATCGGC
SLC2A3 (GLUT3) rev	CAGAGAGACGTGAGCAGCAC

ChIP Primer

Primer	5'-3' Sequence
NICI HRE ChIP fw	TGTGTATGCGTGAGTCTTTGG
NIC I HRE ChIP rev	GAGCCCCGAGCTACATTCCT
EGLN3 HRE fw	AGTGTCCGTTCCCAGCTCAG
EGLN3 HRE rev	TAGGCACAGTAAACAGGCC
SLC2A3 TSS fw.	GCAAGTTTTCTCCACGTCCT
SLC2A3 TSS rev.	AGTCATGATCCCAGCGAGAC

Primers for cloning and sequencing

Primer	5'-3' Sequence
NICI HRE-T2 KpnI	CATGGTACCAAAAGACTCAGCGTGCTGCT
NICI HRE-T2 NheI	CATGCTAGCGCTCTTCCATCCCCAATCTTGT
pGL3 end of vector	CGCTCTCCAATCAAAACAAAA
U6 fw	GGACTATCATATGCTTACCG

Antisense Oligonukleotide

Name	5'-3' Sequence
NEAT1 ASO	mC*mC*mC*mU*mC*T*A*G*T*C*T*T*G*G*C*mU*mC*mA*mU*mU
NICI 1 ASO	mC*mU*mU*mG*mG*A*C*G*C*A*T*T*T*A*C*mC*mC*mA*mA*mA
NICI 2 ASO	mC*mA*mA*mG*mU*G*A*U*C*C*G*A*U*C*U*mC*mU*mU*mC*mG
Scrambled-ASO	mU*mA*mU*mC*mU*G*C*A*C*T*T*C*T*C*T*mC*mA*mC*mA*mC

mN = 2‘-O-MeRNA Base

* = Phosphorothioate backbone

CRISPR sgRNA-guides

Name	5'-3' Sequence
NICI HRE CRISP strand fw	GCGTGCCACACGGCACGTCCGTTTT
NICI HRE CRISP strand rev	GGACGTGCCGTGTGGCACGCCGGTG
NICI CRISPR fw	CGAAGAGATCGGATCACTTGGTTTT
NICI CRISPR rev	CAAGTGATCCGATCTCTTCGCGGTG

Activator sgRNA-guides

Name	5'-3' Sequence
NICI guide#1 fw.	CACCGCGGCGGGTAGCGTGCCACA
NICI guide#1 rev.	AAACTGTGGCACGCTACCCGCCGC
NICI guide#2 fw.	CACCGCCCCCGTGCTGGGATTCCGA
NICI guide#2 rev.	AAACTCGGAATCCCAGCACGGGGGC
NICI guide#3 fw.	CACCGAGCGGCCGGGACGTGCCGTG
NICI guide#3 rev.	AAACCACGGCACGTCCCGGCCGCTC
NICI guide#4 fw.	CACCGCGTGCCACACGGCACGTCC

NICI guide#4 rev.	AAACGGACGTGCCGTGTGGCACGC

siRNA

**Name**	**Sequence**
dHIF sense	CCUACAUCCCGAUCGAUGAtt
dHIF antisense	UCAUCGAUCGGGAUGUAGGtt
HIF-1α sense	CUGAUGACCAGCAACUUGAtt
HIF-1α antisense	UCAAGUUGCUGGUCAUCAGt
HIF-2α sense	CAGCAUCUUUGAUAGCAGUtt
HIF-2α antisense	ACUGCUAUCAAAGAUGCUGtt

Supplementary Figure 1

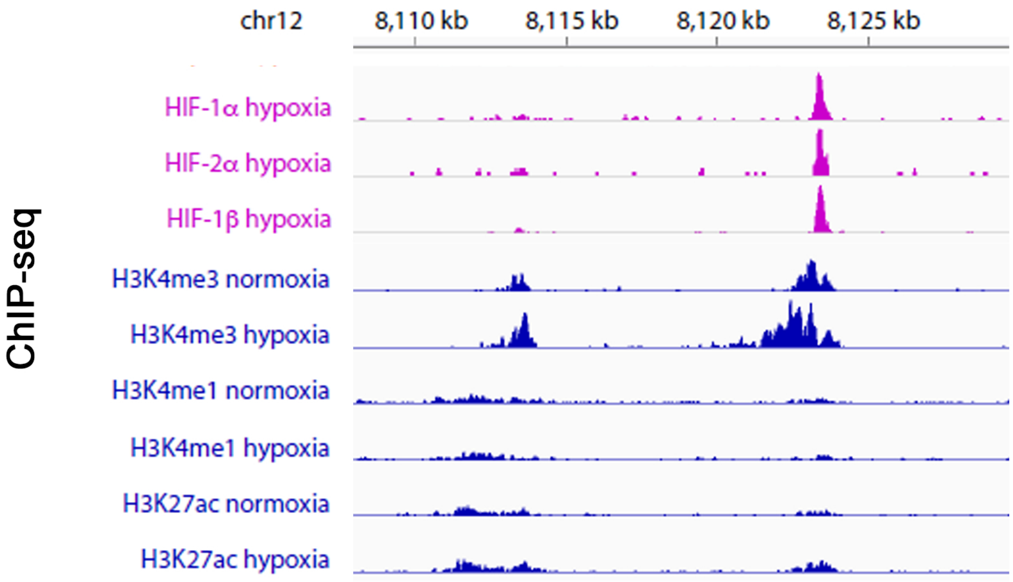

Supplementary Figure 2

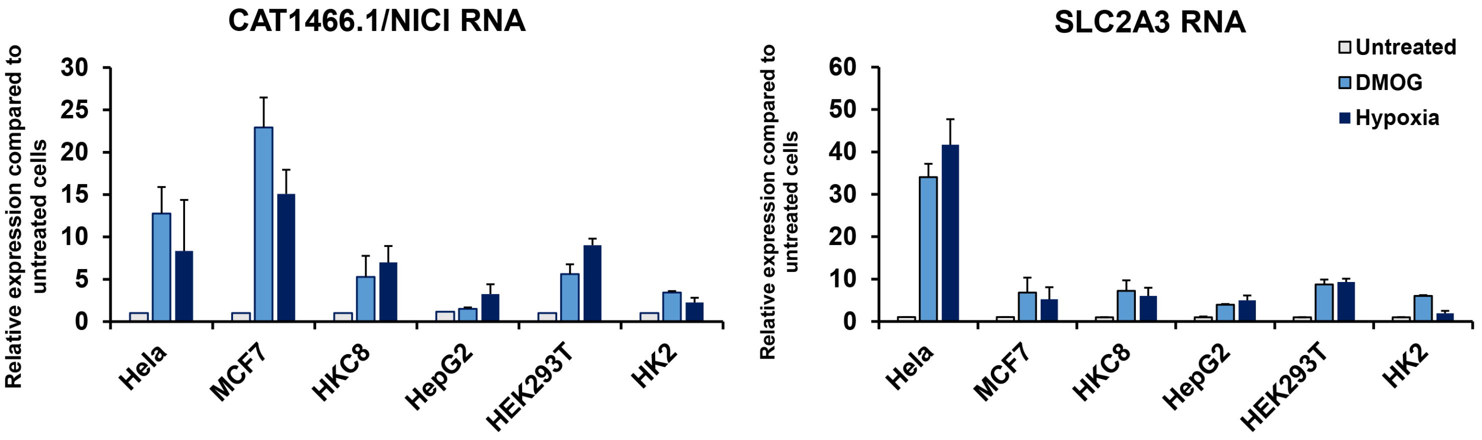

Supplementary Figure 3

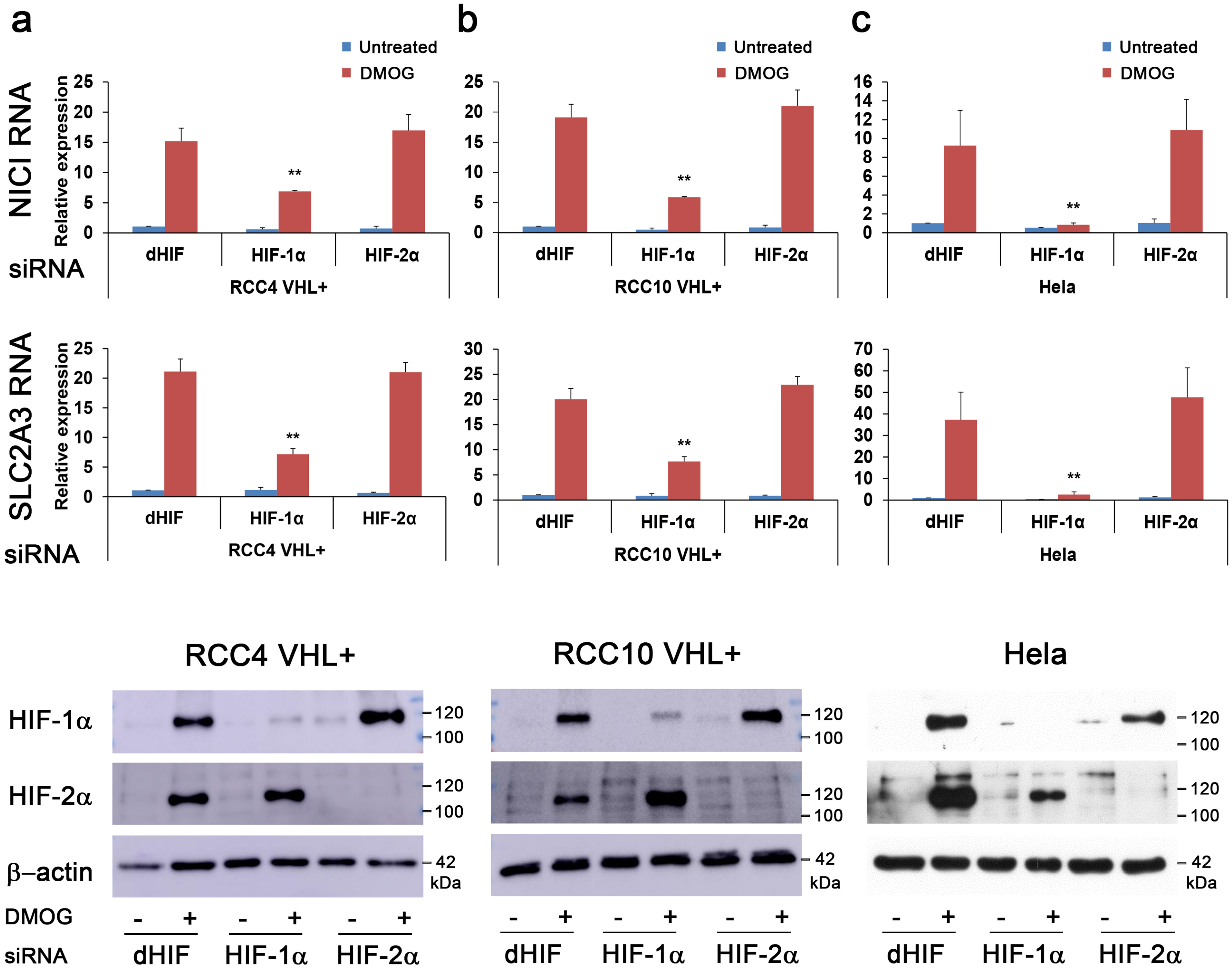

Supplementary Figure 4

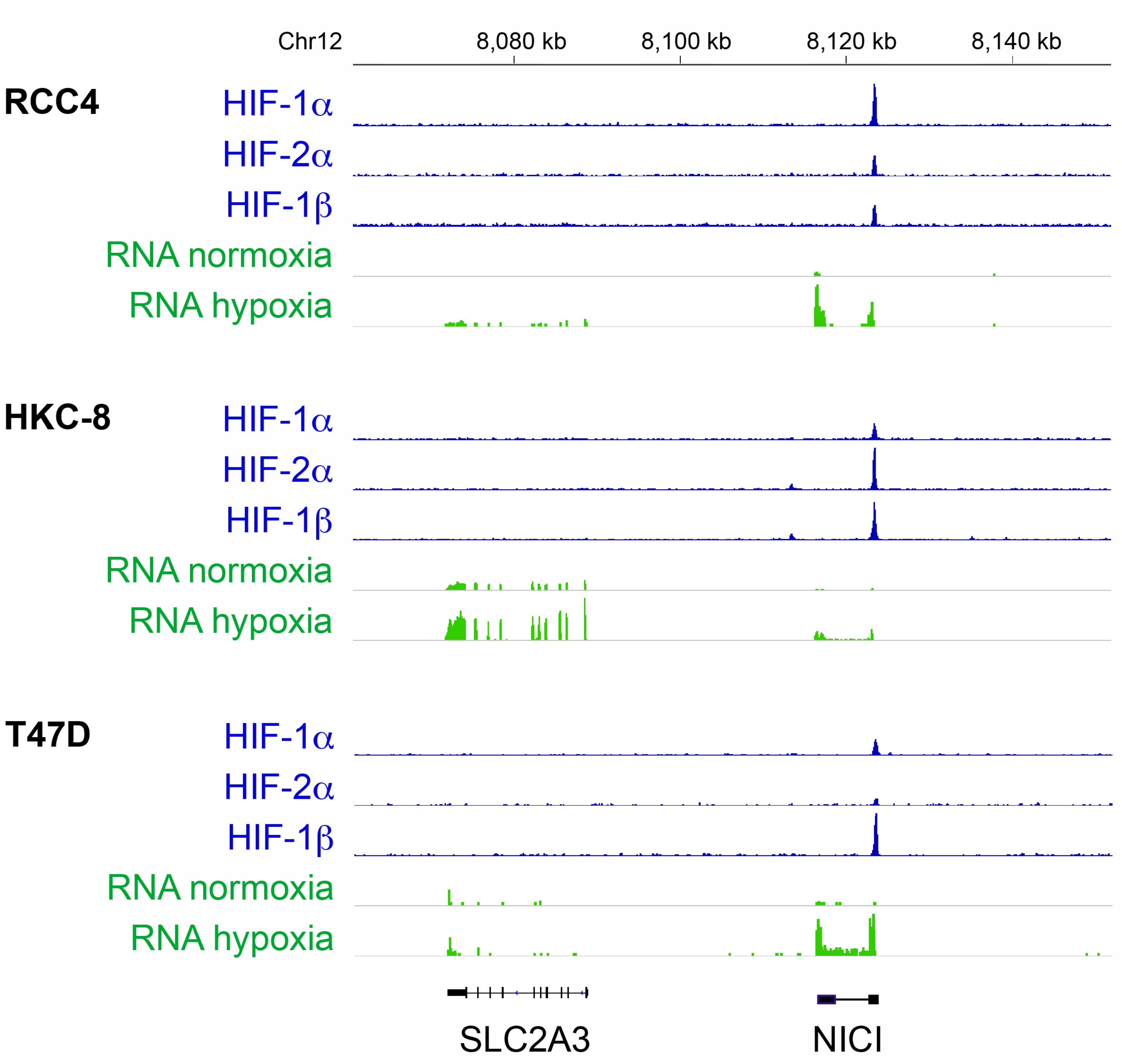

Supplementary Figure 5

a

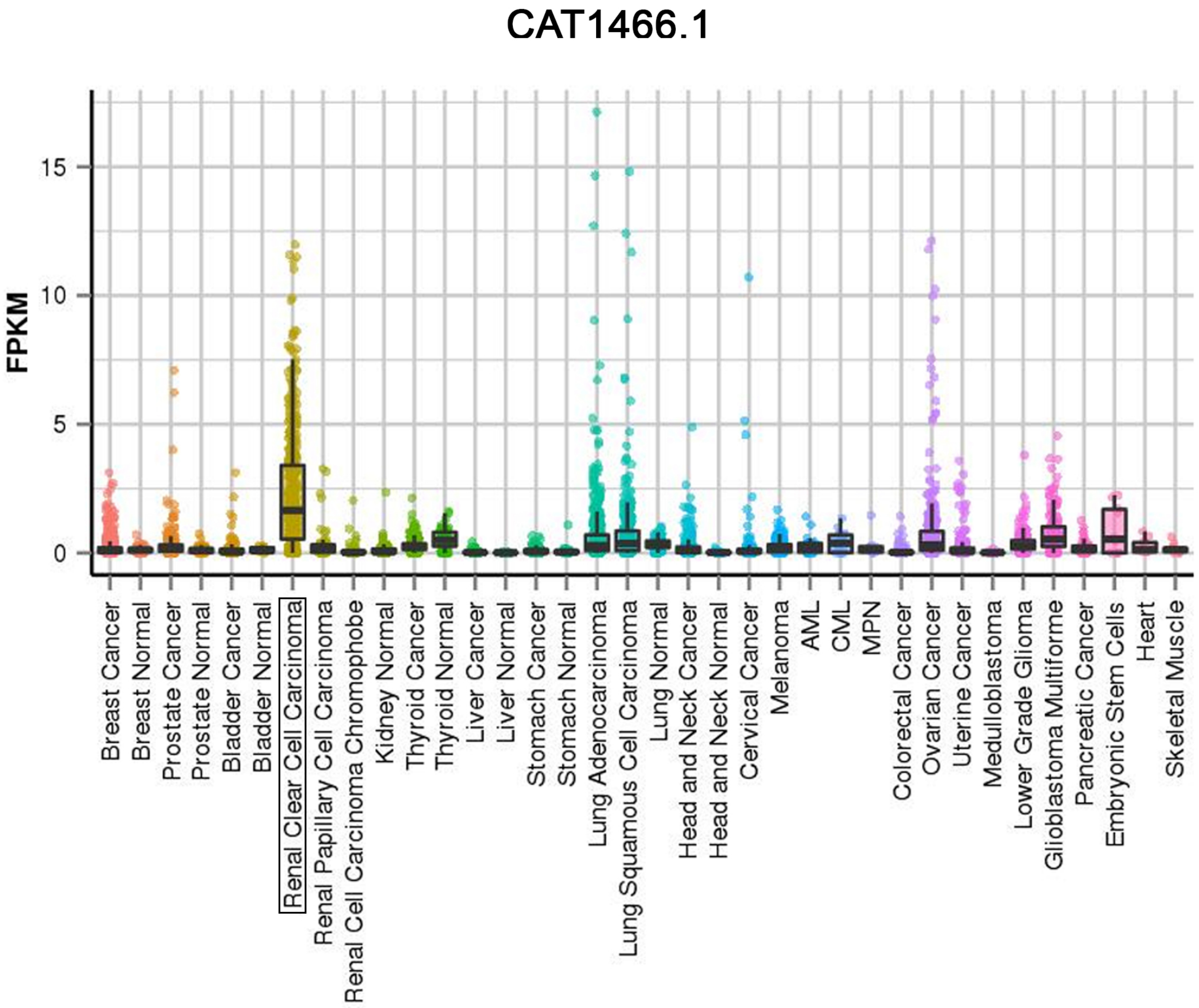

b

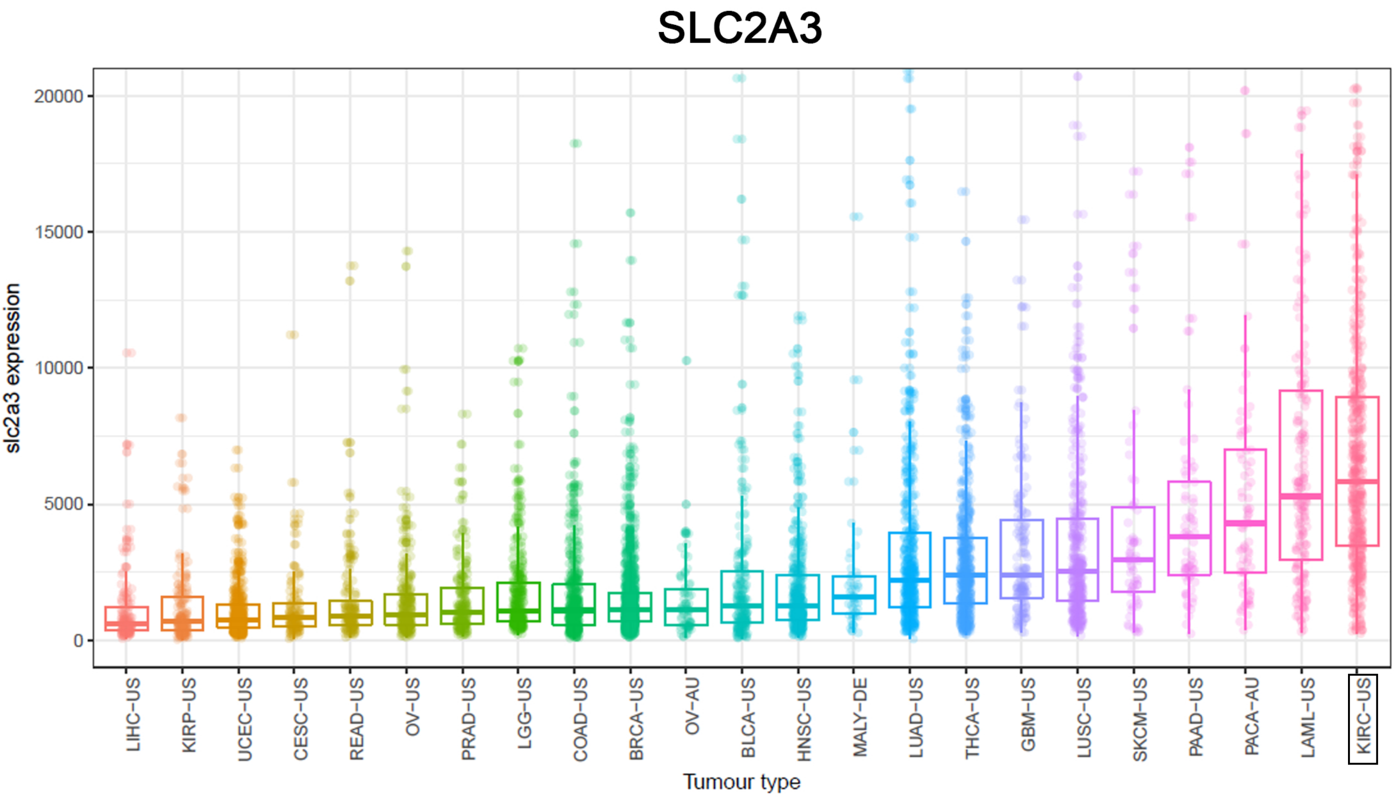

Supplementary Figure 6

a

hg19 Chr12:8,123,206-8,123,563

CAGTCTTTTCATTTTTATTTCTTCCTCTTGGACGCATTTACCCAAATTGG
CCCGAGGGAGGCTGAGACAGCAGATACGAAAGAAAAATGTGTGTATGCGT
GAGTCTTTGGGTGGCGAAGAAGGAGGAGGAGGAATGTAGCTCGGGGCTCG
GCCCCCGTGCTGGGATTCCGAGGGCGGGGGACAGGATGCGCGCGCGGCGG
GTAGCGTGCCACACGGCACGTCCCGGCCGCTGCAGGAAGGCGGGGCTCAG
GTCCCGCCGACTCCCACATGCTGGGGGACACCGGGAGCGCCTCCCACTCC
CAGCCCGCCGGAGAGCCTTGCAATTTTCAGGAATCGGCGTCTTCTCTGGA
AAGCCGCC

b

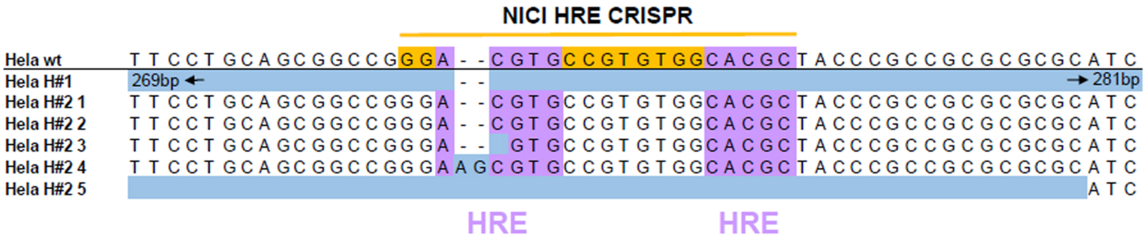

c

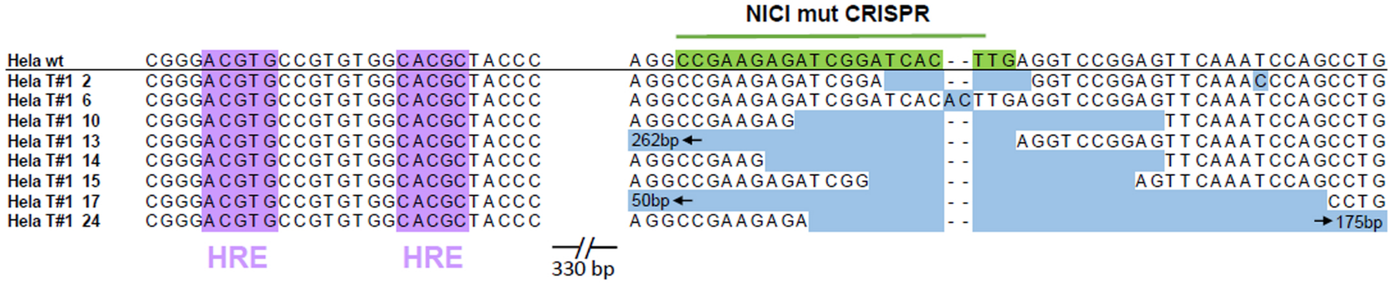

Supplementary Figure 7

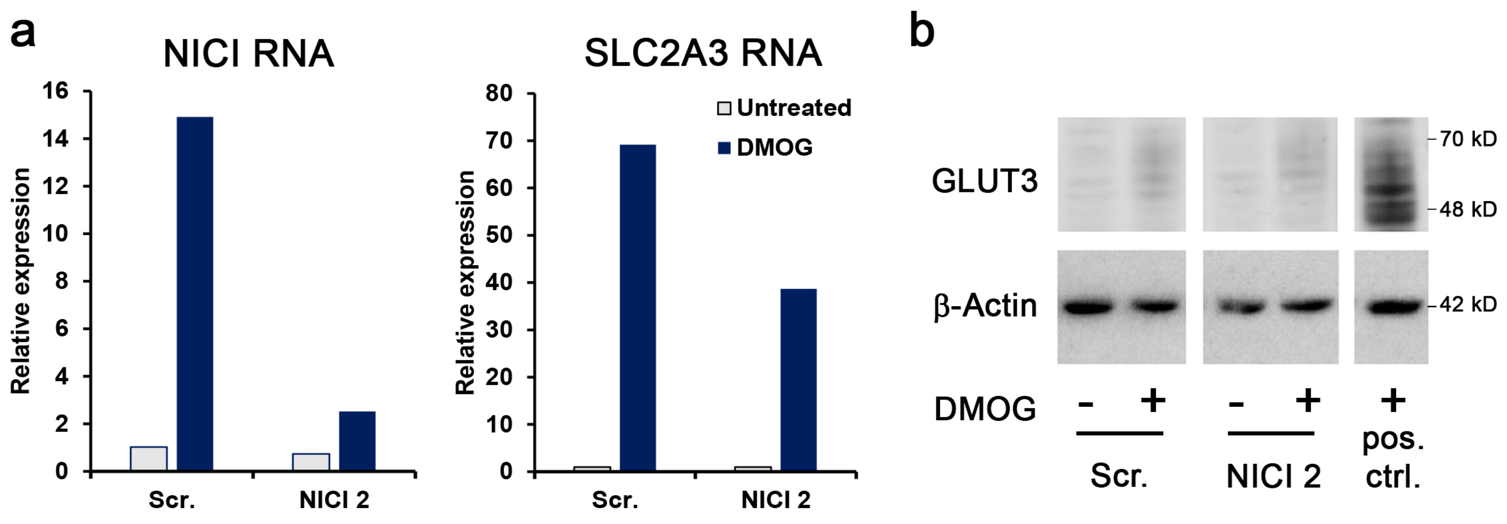

Supplementary Figure 8

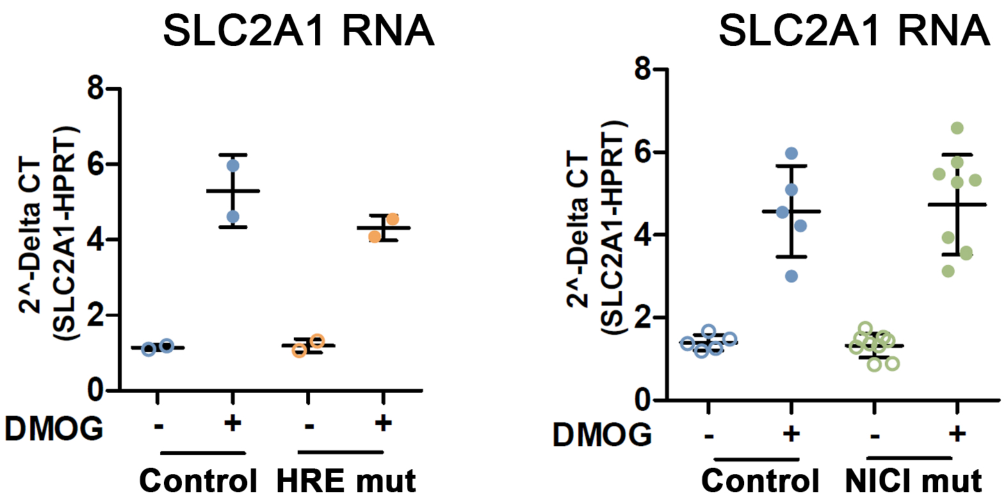

Supplementary Figure 9

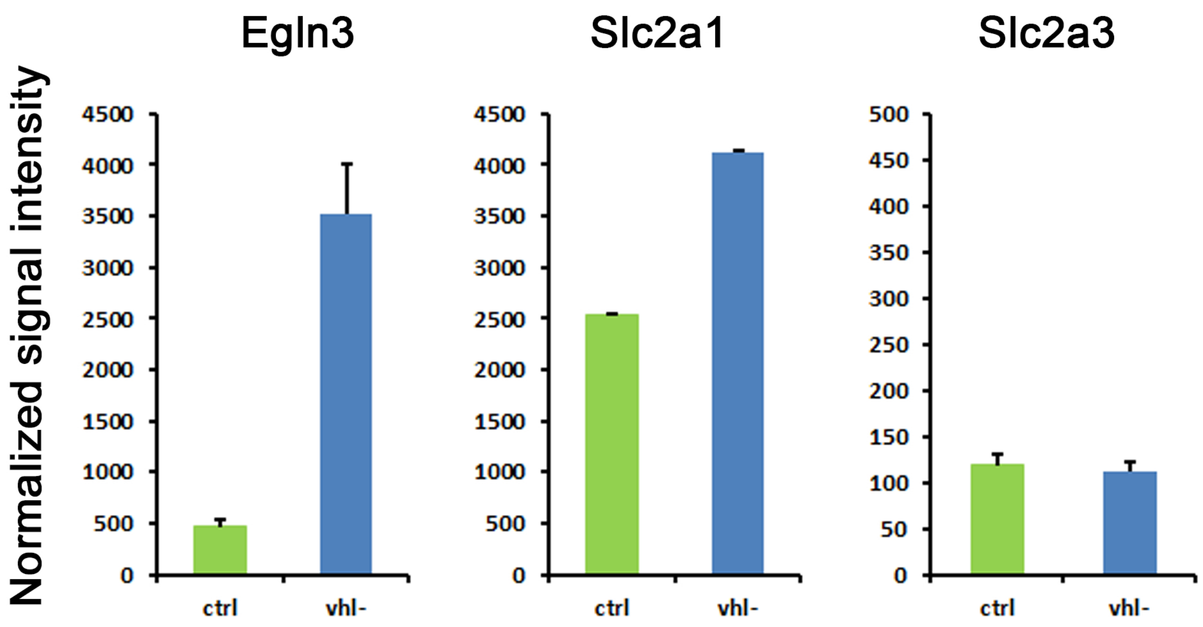

Supplement: Supporting Information [file supp_RA119.009827_153544_1_supp_421695_q08cpx.pdf]
